# Anti-inflammatory effects of potato extract on a rat model of cigarette smoke–induced chronic obstructive pulmonary disease

**DOI:** 10.3402/fnr.v59.28879

**Published:** 2015-10-23

**Authors:** Gui Hua Xu, Jie Shen, Peng Sun, Min Li Yang, Peng Wei Zhao, Yan Niu, Jing Kun Lu, Zhi Qiang Wang, Chao Gao, Xue Han, Lei Lei Liu, Chen Chen Liu, Zhang Yue Cong

**Affiliations:** 1 Department of Clinical Medical Research Center, Inner Mongolia Autonomous Region People's Hospital, Hohhot, Inner Mongolia, China; 2 Department of Neurology, Inner Mongolia Autonomous Region People's Hospital, Hohhot, Inner Mongolia, China; 3 Institute of Microbiology and Immunology, School of Basic Medical Sciences, Inner Mongolia Medical University, Hohhot, Inner Mongolia, China; 4 Inner Mongolia Mengjian Biotechnology company, Wuchua, Inner Mongolia, China; 5 Department of Anatomy, School of Basic Medical Sciences, Inner Mongolia Medical University, Hohhot, Inner Mongolia, China; 6 Department of Forensic Medicine, School of Basic Medical Sciences, Inner Mongolia Medical University, Hohhot, Inner Mongolia, China

**Keywords:** potato extract, cigarette smoke, chronic obstructive pulmonary disease, inflammation

## Abstract

**Highlights**:

(1) Potato extract (PE) exhibits non-toxic effects on mice.

(2) Cigarette smoke (CS)–induced COPD rats exhibit significant thickened and disordered lung markings.

(3) PE could improve the histopathological symptoms of lung tissue in COPD.

(4) PE could increase the expression of IL-10 and reduce the expression of TNF-α and G-CSF in COPD rats.

**Objective:**

This study aimed to evaluate the therapeutic effects of potato extract (PE) on cigarette smoke (CS)–induced chronic obstructive pulmonary disease (COPD).

**Methods:**

PE was first prepared by frozen centrifugation, and its amino acid composition was detected. Toxicity of PE was analyzed by changes in morphology, behavior, routine blood indexes, and biochemical criteria of mice. Then, the COPD rat model was established by CS exposure, and PE, doxofylline, and prednisolone acetate were used to treat these rats. After 45 days of treatment, the morphology and behavior of rats were recorded. In addition, the histopathology of lung tissue was evaluated by chest x-ray and hematoxylin and eosin staining. The expression of interleukine-10 (IL-10), tumor necrosis factor-α (TNF-α), and granulocyte colony-stimulating factor (G-CSF) was detected in serum and lung tissue by enzyme-linked immunosorbent assay (ELISA) and immunohistochemistry, respectively.

**Results:**

Various amino acids were identified in PE, and no toxicity was exhibited in mice. The CS-induced COPD rat model was successfully established, which exhibited significant thickened and disordered lung markings on 90% of the rats. After administering doxofylline and prednisolone acetate, inflammation symptoms were improved. However, side effects such as emaciation, weakness, and loosening of teeth appeared. In the PE group, obviously improved histopathology was observed in lung tissues. Meanwhile, it was revealed that PE could increase the expression of IL-10 and reduce the expression of TNF-α and G-CSF in COPD rats, and doxofylline and prednisolone acetate also elicited similar results.

**Conclusion:**

Our study suggests PE might be effective in the treatment of CS-induced COPD by inhibiting inflammation.

Cigarette smoke (CS) is harmful to human health because of its constituents, including some carcinogens and various toxins such as nicotine and carbon monoxide (1). Nowadays, CS is found to induce diverse functional lung abnormalities and has become one of the most important risk factors for chronic obstructive pulmonary disease (COPD) (2). COPD is an obstructive lung disease characterized by chronically poor airflow, which exhibits the symptoms of breath shortness, cough, and sputum production (3). In clinical settings, COPD seriously affects the quality of life of patients, resulting in high mortality. Approximately 329 million people were affected by COPD around the world in 2010, with 2.9 million fatalities (4, 5). In 2020, COPD is expected to become the fifth leading cause of disease burden and the third leading of mortality (6).

COPD is always associated with increased chronic inflammatory response to hazardous particles or gases in the airways and the lungs (7). When exposed to CS, a mount of inflammatory cells, including macrophages, neutrophils, and lymphocytes, is always accumulated in the lungs. These cells release various inflammatory substances, which could destroy collagen and elastin, stimulate mucosal secretions in lung tissues, and even lead to COPD (1). This process involves various inflammatory factors, including cytokines and chemokines. Therapeutics that target NF-κB activation, including inhibitors of IκB kinases (IKKs), play a major role in treating asthma and COPD (8). As reported, tumor necrosis factor-alpha (TNF-α) was significantly higher in spontaneous sputum at exacerbations and trending higher at the steady state of COPD patients (9). Serum TNF-α concentration was higher in all current smokers than in ex-smokers with COPD (10). Increased IL-6 was mainly associated with smoking burden in patients who had smoked for more than 30 pack-years (11) and was associated with mortality and worse physical performance (12). IL-17A and IL-22 in serum and sputum were higher in COPD patients than healthy smokers and non-smokers, and IL-10 was lower in COPD patients and healthy smokers than non-smokers (13). Moreover, it has been reported that neutralization of granulocyte-macrophage colony-stimulating factor (GM-CSF) could ameliorate COPD and predict its therapeutic utility (14). However, the changes and effects of granulocyte colony-stimulating factor (G-CSF) in CS-induced COPD have not been revealed.

As a starchy tuberous crop, potato is a concentrated source of amino acids, vitamins, minerals, and organic compounds (15). Recently, products made from potatoes, such as kynurenic acid (16) and glycoalkaloids (17), have also been found to be effective in relieving inflammation. It has been reported that potato extract (PE) could alleviate the exacerbation of atopic dermatitis–like skin lesions in mice by suppressing total serum level of IgE and correcting the Th1/Th2 balance (18). The anti-inflammatory effect of potato peel on dextran sulfate sodium–induced colitis was suggested to be associated with suppression of NF-κB and p38 activation in macrophages (19). Evidence also shows that pigmented potato consumption offers the benefit of reducing inflammation and DNA damage in healthy adult males (20). However, no direct anti-inflammatory effects of amino acids derived from potato were revealed. Therefore, it is necessary to determine the distinctive role of potato products in COPD and also investigate the uncertain effect of potato on CS-induced COPD.

In this study, the therapeutic effects of PE were investigated on a rat model of CS-induced COPD in terms of both histopathological and immunological changes (TNF-α, IL-10, and G-CSF) in lung microenvironment.

## Materials and methods

All experimental animal procedures were conducted according to the Institutional Animal Care and Use Committee at Inner Mongolia Medical University (Hohhot, Inner Mongolia, China). All of our study protocols were approved by the Ethics Committee of Inner Mongolia Medical University before the commencement of the study, and we were given permission to conduct the study.

### PE and amino acid analysis

Fresh potato Kexing IV was developed by the Potato Research Institute of Heilongjiang Academy of Agricultural Sciences (Heilongjiang, China), and systematically cultivated by Sheng Feng Potato Industry Planting Base in Wuchua area of the Inner Mongolia Autonomous Region in China. The PE was prepared by frozen centrifugation, which has been accorded the national patented invention (No. ZL200810091883.6). In brief, potato was first frozen at −30°C for 12 h, then thawed at 35°C, and disrupted. After centrifuging at 4,000 rpm for 30 min, the supernatant was collected. Then the extracted liquid was purified by macroporous adsorptive resins (0.45 µm, ChiCheng Pharmaceutical Technology, Zhejiang, China) at room temperature, and stored at 4°C until tested.

Amino acid composition of the PE was detected by an amino acid analyzer (L-8900, Hitachi, Tokyo, Japan). Different amino acids were eluted sequentially based on their ionic strength. (Acidic amino acids were obtained first, then neutral amino acids, and finally, basic amino acids.) Then, these amino acids were reacted with ninhydrin at 135°C. Finally, the concentration of amino acids was quantified by an ultraviolet detector (VIS-7220, Ruili, China) at 570 and 440 nm.

### Toxicity test of PE

Toxicity of the PE was analyzed by acute oral toxicity test. A total of 20 healthy Kunming mice (10 males and 10 females; age: 6–8 weeks; weight: 18–22 g; purchased from Beijing Weitong Lihua Experimental Animal Technology, Beijing, China) were used to determine the maximum tolerated dose. After fasting for 16 h, 15 g/kg body weight (BW) PE was administered to mice by oral gavage. Then, normal diet was given to all these subjects, and toxicity signs and the death rate were recorded in the following 2 weeks.

On the contrary, different concentrations of PE were further used to identify the long-term toxicity of PE in 90 days. A total of 80 healthy Kunming mice (40 males and 40 females) were randomly divided into four groups according to the concentrations of PE: 0.0/kg BW (negative control), 0.5 g/kg BW, 5 g/kg BW, and 10 g/kg BW. Each of mice was kept in single cage with free access to food and water (feed replenished once or twice a week). During 90 days, the weight, food consumption, morphological characteristics, behavior, and death state of these mice were recorded once daily. In addition, blood sample was collected from the femoral artery of mice on Day 45 and 90. Routine blood indexes, including hemoglobin, blood cell count, white blood cell classification, were detected by a blood analyzer (MEK-6450K, Shanghai Photoelectric Medical Instrument, Shanghai, China), and multiple biochemical criteria (fasting for 16 h before detection), including alanine aminotransferase, aspartate aminotransferase, blood urea nitrogen creatinine, and blood glucose, were detected by a biochemical analyzer (7020, Hitachi High-Technologies, Shanghai, China). In addition, the weight of the viscera, including the liver, spleen, and kidney, was also measured. Moreover, the histopathology of the viscera was observed by a microscope (DM4000B Leica, Wetzlar, Germany).

### Establishment of CS-induced COPD rat and drug treatment

A total of 110 Sprague Dawley (SD) rats, including 55 males and 55 females (Beijing Weitong Lihua Experimental Animal Technology), were exposed to either fresh air (normal group) or CS generated by Daqingshan cigarettes (Tar: 12.5, Hohhot Cigarette Factory, Hohhot, China; model group). In the model group, the rats were completely exposed to smoke daily, four cigarettes per day for 2 months (twice a day in the morning and night). During this period, the morphology, behavior, and death rate were recorded. In addition, lung tissue morphology and lung markings were both evaluated by chest x-ray film (Editormp601, Berlin, Germany). The characteristics such as obviously thickened and disordered lung markings were used as the criteria for COPD.

The CS-induced COPD rat model was intragastrically treated with 10 mg PE (*n*=30), 16.3 mg doxofylline (*n*=10; Heilongjiang Fuhehuaxing Pharmaceutical, Heilongjiang, China), and 1.64 mg prednisolone acetate (*n*=10; Zhejiang Xianju Pharmaceutical, Zhejiang, China) each day for 45 days. COPD rats without any treatment were considered to be the control group (*n*=10).

### ELISA assessment of IL-10, TNF-α, and G-CSF

After 45 days of treatment, the rats in different groups were anaesthetized by chloral hydrate (0.33 ml/mg BW) and blood samples were collected from the heart. These blood samples were first stored at 4°C for 2 h, and then the serum was separated by centrifugation at 2,500 rpm/min for 10 min. The concentrations of serum IL-10, TNF-α, and G-CSF were measured using quantitative ELISA kits (R&D Systems, Minneapolis, MN) following the manufacturer's instructions. The serum samples were added into 96-well microtiter plates and incubated for 30 min at 37°C with anti-rat IL-10, TNF-α, and G-CSF in coating buffer at a dilution of 1:100. Then, the wells were washed five times with PBS before adding peroxidase-labeled biotinylated secondary antibodies. After 30 min of induction at 37°C, the plates were treated with TMB substrate solution for 10 min, and the reaction was stopped by the addition of TMB stop solution. Finally, the optical density (OD) was measured at 450 nm by a microplate reader (1510, Thermo Fisher Scientific, Finland).

### Histological examination

After 45 days of treatment, lung tissue was removed from the rats of different groups for histological analysis. Paraformaldehyde fixing solution (4%) was infused into the lungs, and bronchus specimens were dehydrated and embedded in paraffin. For histological examination, 4-µm sections of embedded tissues were deparaffinized and stained with hematoxylin and eosin (HE). Also, deparaffinized lung tissue sections were processed for antigen retrieval using target retrieval solution (pH 9.0; Dako, Cupertino, CA) at 120°C for 4 min. Non-specific immunoreactions were blocked at room temperature for 30 min using the Protein-Block kit (Dako). After washing with PBS, tissue sections were incubated overnight at 4°C with primary antibodies such as anti-rat IL-10, TNF-α, and G-CSF (1:100, R&D Systems). Positive reactions were visualized following 3,3′-diaminobenzidine tetrahydrochloride (Dako) reaction. Nuclear counter stainings were conducted with hematoxylin. The images of HE and immunohistochemistry were randomly taken using a microscope (DM4000B Leica).

### Statistical analyses

All data were expressed as mean±s.e.m. Statistical analysis was performed by SPSS version 17.0 (SPSS Inc., Chicago, IL). The difference between different groups was determined by one-way ANOVA. A *P*-value less than 0.05 was considered to be significantly different.

## Results

### Amino acid compositions of PE

Total content of amino acids was 648.3 mg/ml in PE, which comprises various amino acids, including aspartic acid (227.348 mg/100 ml), glutamic acid (127.686 mg/100 ml), valine (44.407 mg/100 ml), alanine (25.295 mg/100 ml), threonine (23.350 mg/100 ml), leucine (22.354 mg/100 ml), isoleucine (21.290 mg/100 ml), phenylalanine (21.909 mg/100 ml), glycine (15.796 mg/100 ml), serine (15.620 mg/100 ml), cystine (9.005 mg/100 ml), methionine (12.986 mg/100 ml), lysine (24.309 mg/100 ml), arginine (20.140 mg/100 ml), proline (21.162 mg/100 ml), and histidine (8.583 mg/100 ml).

### Toxicity of PE

During 2 weeks of administration of 15.0 g/kg BW PE, no obvious abnormality in general performance and behavior of mice were detected, no significant differences of body weight and food consumption were established, and no death occurred, suggesting that maximum tolerated dose (MTD) for all rats was above 15.0 g/kg BW PE.

During the 90-day period when the mice were fed with different concentrations of PE, the growth, development, and behavior of the mice were normal, with no fatality in each group. In addition, no significant differences were found in body weight and food consumption (*P*>0.05). Both routine blood indexes and multiple biochemical criteria were relatively the same in each group (*P*>0.05). In addition, viscera weight was similar in each group (*P*>0.05), and no histopathological injury was exhibited on the viscera (data were not shown).

### CS-induced COPD in rat

In order to establish the model of COPD, rats were exposed to CS for 2 months. As a result, cough was first detected in 15% of rats in the first 2 weeks. Then the rate of cough reached 40% in the third week, and laryngeal stridor began to appear in 10% of rats. After 1-month treatment, all rats exhibited the symptoms of cough (some rats had continuous cough), and 50% of rats exhibited the symptoms of laryngeal stridor with considerable nasal secretion. In addition, reduced weight, flagging spirit, and hypokinesia were also observed in these rats. Chest x-ray showed significantly thickened and disordered lung markings in 60% of rats. Two months later, 90% of rats had significantly thickened and disordered lung markings, suggesting that the model of COPD was successfully established. During this process, there were three fatalities because of severe pulmonary infection.

### Effects of PE on COPD rats

**Fig. 1:**
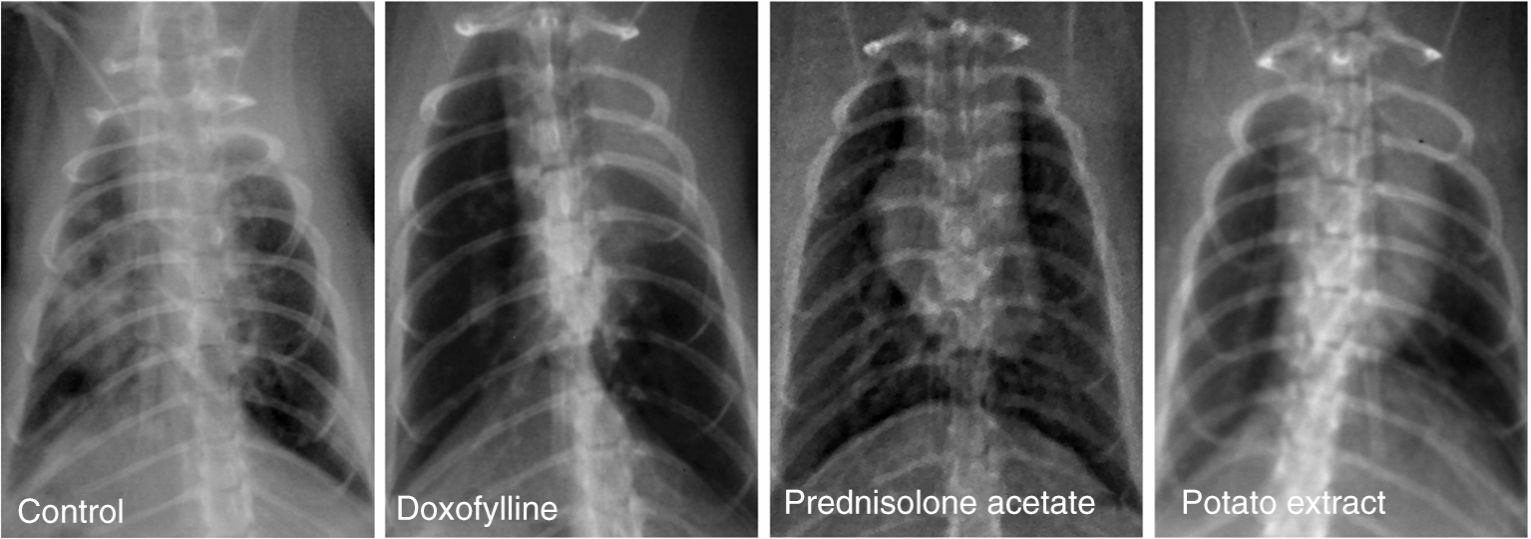
Chest x-ray film observation of lung tissue in COPD rats after treatment by potato extract (*n*=10), doxofylline (*n*=10), and prednisolone acetate (*n*=10) for 45 days. COPD rats without any treatment were defined as the control group (*n*=10).

After 45 days of treatment, the morphology and behavior of COPD rats were evaluated. As shown in Fig. 1, significant histopathological changes were observed in the lungs of COPD rats, such as thickened and disordered lung markings; high-density patchy, cloudy shadows; and blurred edges at the lower lobe of the right lung. In the doxofylline and prednisolone acetate group, these inflammation symptoms were significantly lessened, with no obvious, substantial infiltration and less thickened, disordered lung markings. However, some rats in the doxofylline group were considered to be thin and weak, characterized by loosening of teeth due to pulmonary encephalopathy after treatment. In addition, although the pesticide effects of prednisolone acetate were better than doxofylline, side effects such as central obesity and swollen tongue were always found in the prednisolone acetate group. Encouragingly, obviously improved symptoms without side effects were exhibited in the PE group, including absence of histopathological changes on lung tissue, sleek and bright pelage, increased weight, and normal behavior. Meanwhile, clear lung markings and non-existent substantial infiltration were also observed in lung tissue by chest x-ray (Fig. 1). On the contrary, no significant differences were observed on the death rate of these three groups (about 20%) during treatment.

**Fig. 2:**
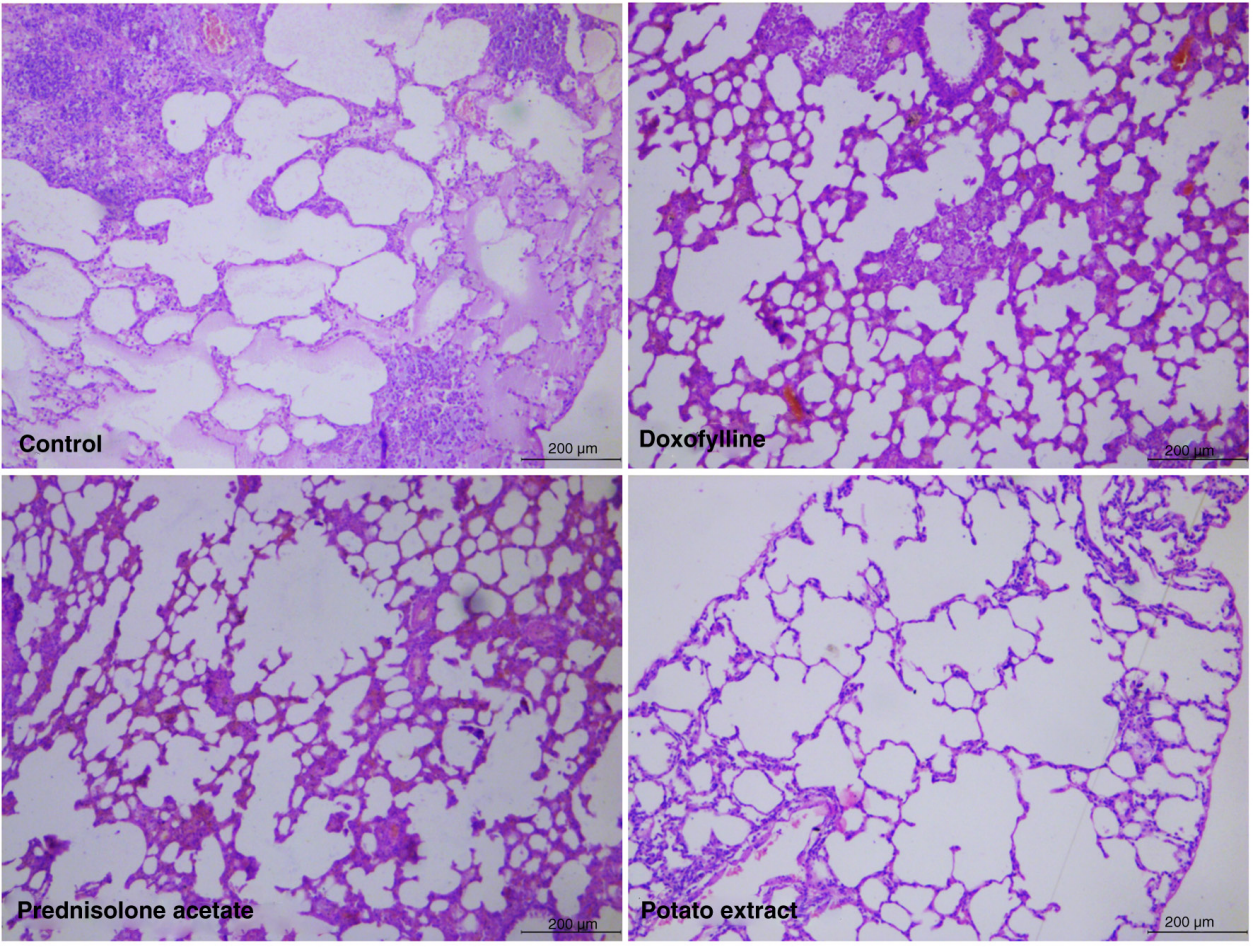
Hematoxylin and Eosin (HE) staining of lung tissue in COPD rats after treatment by potato extract, doxofylline, and prednisolone acetate for 45 days. COPD rats without any treatment were defined as the control group. Scale bar=200 µm.

To further evaluate the pathological changes in the lung tissue, HE staining was performed on each group. As a result, the lung tissue was significantly damaged in the rat model of COPD, in which there were degeneration and necrosis of bronchial mucosal epithelium cells, inflammatory cell infiltration in bronchial wall, serous fluid exudation from alveolar space, and pulmonary consolidation (Fig. 2). However, the degree of inflammatory cell infiltration, serous fluid exudation, and the area of consolidation were significantly reduced by the administration of doxofylline and prednisolone acetate (Fig. 2). Furthermore, the histomorphology of the lung tissue in the PE group was revealed to be relatively normal without infiltration, indicating PE is more effective in the treatment of COPD compared with both doxofylline and prednisolone acetate (Fig. 2).

### Effects of PE on the expression of IL-10, TNF-α, and G-CSF

**Fig. 3:**
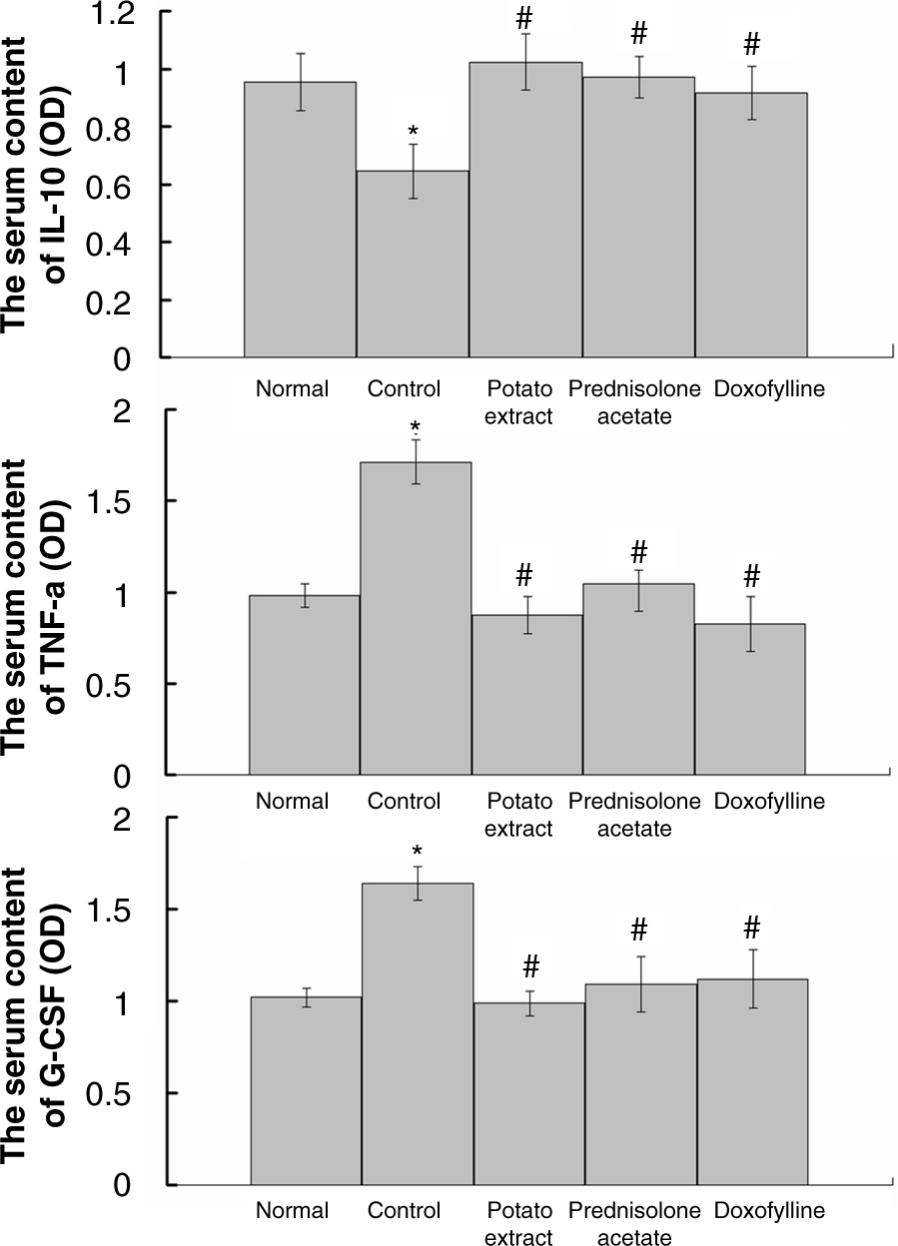
Serum contents of interleukine-10 (IL-10), tumor necrosis factor-α (TNF-α), and granulocyte colony-stimulating factor (G-CSF) in COPD rats after treatment by potato extract, doxofylline, and prednisolone acetate for 45 days. COPD rats without any treatment were defined as the control group. Normal rats without exposure to cigarette smoke were enrolled in the normal group. *P<0.05 vs the normal group; *^#^P*<0.05 vs the controll group.

**Fig. 4:**
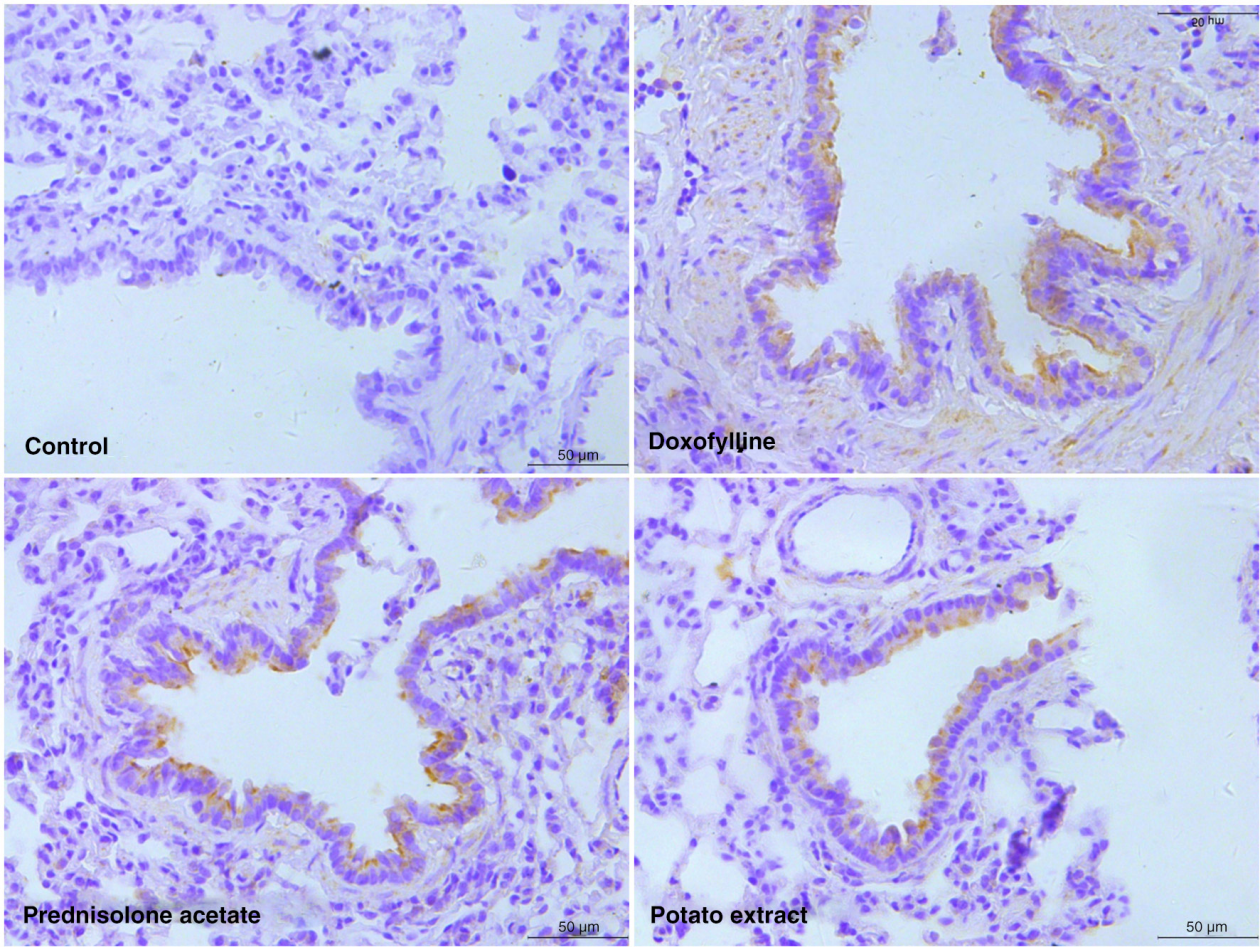
Immunohistochemistry of tumor necrosis factor-α (TNF-α) in lung tissue of COPD rats after treated by potato extract, doxofylline, and prednisolone acetate for 45 days. COPD rats without any treatment were enrolled in the control group. Scale bar=50 µm.

**Fig. 5:**
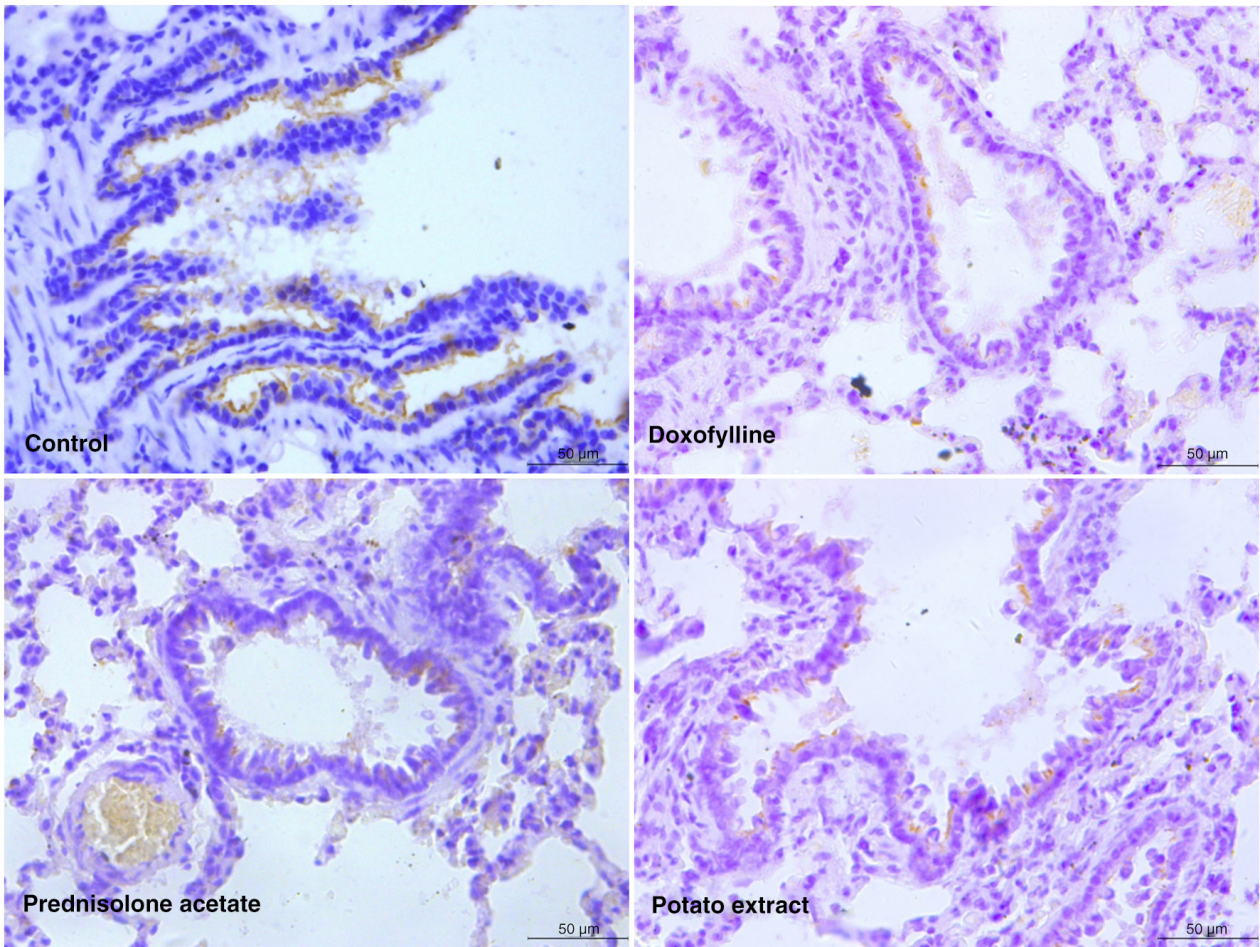
Immunohistochemistry of granulocyte colony-stimulating factor (G-CSF) in lung tissue of COPD rats after treatment by potato extract, doxofylline, and prednisolone acetate for 45 days. COPD rats without any treatment were enrolled in the control group. Scale bar=50 µm.

**Fig. 6:**
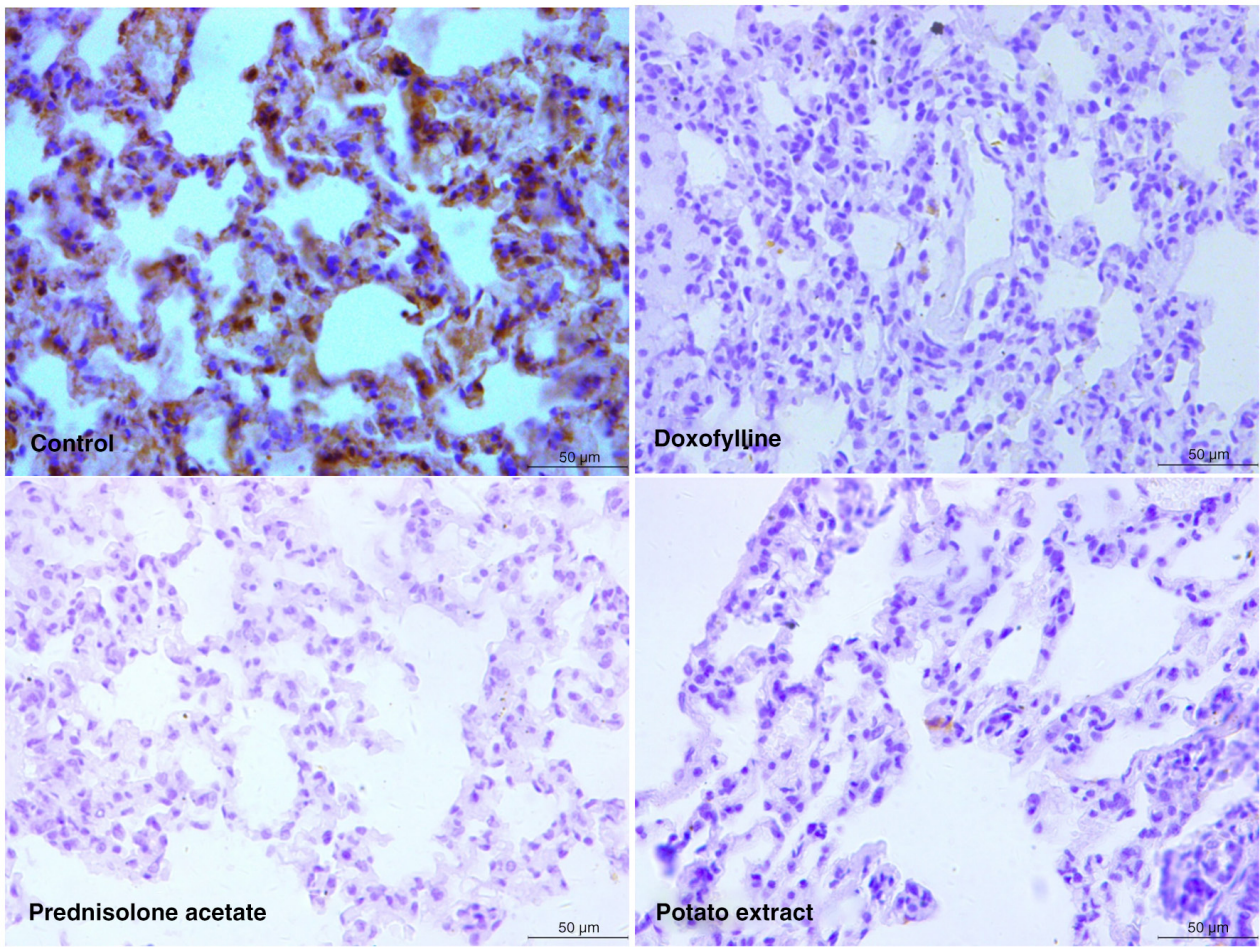
Immunohistochemistry of IL-10 in lung tissue of COPD rats after treated by potato extract, doxofylline, and prednisolone acetate for 45 days. COPD rats without any treatment were enrolled in the control group. Scale bar=50 µm.

To evaluate the inflammatory changes in COPD rats in response to different drugs, the expression of IL-10, TNF-α, and G-CSF was analyzed. As a result, significantly reduced expression of IL-10 and increased expression of TNF-α and G-CSF were induced after CS treatment (*P*<0.05), which could be observed in both serum (Fig. 3) and lung tissue (Figs. 4–6). However, the expression of IL-10, TNF-α, and G-CSF recovered to normal levels by the administration of doxofylline and prednisolone acetate. Furthermore, PE was also found to be effective in the reduction of inflammation, and it induced similar expression levels of IL-10, TNF-α, and G-CSF compared with both doxofylline and prednisolone acetate (Figs. 3–6).

## Discussion

The COPD is a common and serious respiratory disease, which has a high risk of mortality (21). This disease is characterized by continued airflow limitation and is considered to be closely related to inflammatory response (3). Until now, various drugs, including chemical compounds and natural plants, such as 1,8-cineole (22) and resveratrol (23), have been used to treat COPD. However, the therapeutic efficacy is diverse and limited (24, 25). In this study, PE was found to increase the expression of IL-10 and to reduce the expression of TNF-α and G-CSF in the rat model of COPD; the curative effects of PE in lung inflammation are similar to that of doxofylline and prednisolone acetate.

CS-induced rat model has always been used to evaluate drug effects on lung inflammation, as it exhibits physiological effects akin to humans (26, 27). In line with previous studies, it has been reported that the 2-week CS exposure was enough to induce alveolar wall destruction and airspace enlargement in mice (28, 29). Also, rats exposed to CS for 2 months exhibited significantly thickened and disordered lung markings, suggesting CS-induced COPD model was successfully established.

As indicated, both doxofylline and prednisolone acetate can inhibit lung inflammation and are considered to be common therapy drugs for COPD with reliable evidence from clinical trials (30, 31). Therefore, doxofylline and prednisolone acetate were used as positive controls in this study to evaluate the therapeutic effects of PE on CS-induced COPD rats. Pathological changes of COPD often occurred in the airway wall and lung parenchyma. In COPD rats, significant inflammation of the lung parenchyma was observed by chest x-ray, including thickened and disordered lung markings; high-density patchy, cloudy shadows; and blurred edges at the lower lobe of the right lung. After treatment with doxofylline and prednisolone acetate, these pathological changes were significantly lessened, although some side effects were always present in these rats. On the contrary, by lessening the inflammatory pathological changes in lung tissue without any detectable side effects, the PE exhibited its superior therapeutic effects compared with both doxofylline and prednisolone acetate. In addition, inflammatory cell infiltration and increased mucus secretion always occurred in the airway wall in the case of COPD (7). In this study, HE staining of bronchiolar epithelium showed that the degree of inflammatory cell infiltration, serous fluid exudation, and consolidation area were significantly reduced by the administration of doxofylline and prednisolone acetate. Moreover, PE was considered to be more effective in relieving bronchiolar inflammation. To sum up, PE could protect the lung parenchyma and airway wall by reducing the inflammatory response to CS.

Recently, a variety of cytokines and chemokines have been identified to be involved in CS-induced lung inflammation (32). For example, CS could induce high expression of TNF-α in the lung tissue of normal mice (33), and IL-10 in serum and sputum were lower in COPD patients and healthy smokers than non-smokers (13). In this study, the alteration of the TNF-α, IL-10, and G-CSF was investigated for an in-depth evaluation of the inflammatory changes of lung tissue in COPD and the therapeutic effect of PE. TNF-α was an important pro-inflammatory cytokine involved in immunoregulation and inflammation. It has been reported that TNF-α could mediate trauma-induced cell inflammation by stimulating the release of cytokines such as IL-4 and IL-6 and finally lead to severe inflammatory lesions on tissue and organs (34). On the contrary, IL-10 was as an anti-inflammatory cytokine, which could not only inhibit the synthesis of pro-inflammatory cytokines such as IFN-γ, IL-2, IL-3, TNFα, and GM-CSF but also enhance B-cell survival, proliferation, and antibody production (35). Notably, PE was found to increase the expression of IL-10 and reduce the expression of TNF-α in serum and lung tissues of CS-induced COPD rats, which indicated that the inflammatory process was significantly mitigated by PE. The expression of G-CSF was found to be reduced in PE-treated COPD rats in this study. As a glycoprotein, G-CSF was able to stimulate the production, proliferation, and differentiation of neutrophils through various signal pathways, including Janus kinase (JAK)/signal transducer and activator of transcription (STAT), Ras/mitogen-activated protein kinase (MAPK), and phosphatidylinositol 3-kinase (PI3K)/protein kinase B (Akt) (36, 37). When pro-inflammatory cytokines were activated, oxygen-free radicals and lysosomal enzymes were always released by these neutrophils causing inflammation (38). The reduced expression of G-CSF by PE further illustrated that the inflammatory process in COPD was relieved. To sum up, we suspect PE was able to relieve inflammation of lung tissue by inhibiting TNF-α and G-CSF, activating IL-10, and thereby effectively treating CS-induced COPD.

PE comprises various amino acids, vitamins, minerals, and organic compounds. It has been reported that nightshade in potato is one of the most effective components in anti-inflammation (19). However, amino acids and vitamins only exhibit auxiliary effects on inflammation and no direct anti-inflammatory effects were revealed. Therefore, some other effective medical components and anti-inflammatory mechanisms of PE still need to be studied.

In conclusion, PE exhibits significant anti-inflammatory effects on the lung tissue of CS-induced COPD rats, and it may be a promising therapeutic drug for COPD.
